# P01-001 – Musculoskeletal sonography in FMF patients

**DOI:** 10.1186/1546-0096-11-S1-A5

**Published:** 2013-11-08

**Authors:** O Karadag, S Yilmaz, V Yazisiz, M Cinar, H Erdem, S Pay, A Dinc

**Affiliations:** 1 Division of Rheumatology, Gulhane School of Medicine, Ankara, Turkey

## Introduction

Familial Mediterranean fever (FMF) is an autoinflammatory disorder characterized by recurrent febrile polyserositis and musculoskeletal findings. Chronic arthritis and/or accompanying seronegative spondyloarthropathy have been reported in FMF. But little is known about musculoskeletal changes during attack free period.

## Objectives

This study is aimed to investigate the musculoskeletal sonographic changes during attack free period.

## Methods

Totally 29 consecutive FMF male patients at attack free period and 17 male controls were included into the study. Physical examination (PE) was performed to detect Achilles enthesitis and/or retrocalcaneal bursitis, knee arthritis. US of the lower extremity were performed bilaterally. Grey-scale (GS) and power Doppler (PD) scores on a 0–2 semi-quantitative scale (0: no, 1: mild, 2: moderate, 3: marked).

## Results

Mean ages of patients and controls were 24.3 ±4.9, 25.8± 5.5 years (p=0.338). Fourteen (48.2%) of FMF patients had reported arthralgia and/or arthritis during attacks. In 7 of 15 patients who had not reported joint problems, a sonographic pathology was found (4 of them was mild effusion at knee and 3 of them was retrocalcaneal bursitis and medial tenosynovitis. No PDS was found in knee or ankle joint. But there was no pathology in controls.

## Conclusion

Even though none of the FMF patients had chronic arthritis, an increased rate of mild effusion and tenosynovitis was found. This finding might reflect an increased rate of musculoskeletal finding compared to healthy controls.

## Disclosure of interest

None declared

